# Investigation of the variants at the binding site of inflammatory transcription factor NF-κB in patients with end-stage renal disease

**DOI:** 10.1186/s12882-019-1471-2

**Published:** 2019-08-05

**Authors:** Jia-Hwa Yang, Wei-Teing Chen, Meng-Chang Lee, Wen-Hui Fang, Yu-Juei Hsu, Chin-Lin, Hsiang-Cheng Chen, Hsueh-Lu Chang, Chien-Fu Chen, Min-Yu Tu, Chien-Wei Kuo, Yuan-Hau Lin, Po-Jen Hsiao, Sui-Lung Su

**Affiliations:** 1 School of Public Health and Graduate institute of Life Sciences, National Defense Medical Center, No.161, Sec. 6, Minquan E. Rd., Neihu Dist., Taipei City, 114 Taiwan, Republic of China; 2 Division of Chest Medicine, Department of Medicine, Cheng Hsin General Hospital, Taipei, Taiwan, Republic of China; 3 Department of Medicine, Tri-Service General Hospital, National Defense Medical Center, Taipei, Taiwan, Republic of China; 4 Institute of Preventive Medicine, National Defense Medical Center, Taipei, Taiwan, Republic of China; 5 Department of Family and Community Medicine, Tri-Service General Hospital, Taipei, Taiwan, Republic of China; 6 Division of Nephrology, Department of Medicine, Tri-Service General Hospital, Taipei, Taiwan, Republic of China; 7 School of Public Health, National Defense Medical Center, Taipei, Taiwan, Republic of China; 8 Division of Rheumatology/Immunology/Allergy, Department of Internal Medicine, Tri-Service General Hospital, National Defense Medical Center, Taipei, Taiwan, Republic of China; 9 Department of Orthopedics, Taichung Armed Forces General Hospital, Taichung, Taiwan, Republic of China; 10 Department of Orthopedics, Kaohsiung Armed Forces General Hospital, Gangshan Branch, Kaohsiung, Taiwan, Republic of China; 11 Division of Nephrology Dialysis, Shih-Kang Clinic, New Taipei City, Taiwan, Republic of China; 12 Division of Nephrology Dialysis, Yuan-Lin Clinic, Taipei, Taiwan, Republic of China; 13 Division of Nephrology, Department of Internal Medicine, Tri-Service General Hospital, National Defense Medical Center, Taipei City, Taiwan, Republic of China; 14 Division of Nephrology, Department of Internal Medicine, Taoyuan Armed Forces General Hospital, Taoyuan City, Taiwan, Republic of China; 15 Big Data Research Center, Fu-Jen Catholic University, Taipei, Taiwan, Republic of China; 16 Department of Life Sciences, National Central University, Taoyuan City, Taiwan, Republic of China

**Keywords:** Nuclear factor-kappa B (NF-κB), End-stage renal disease (ESRD), Single nucleotide polymorphisms (SNPs)

## Abstract

**Background:**

A chronic inflammatory state is a prominent feature in patients with end-stage renal disease (ESRD). Nuclear factor-kappa B (NF-κB) is a transcription factor that regulates the expression of genes involved in inflammation. Some genetic studies have demonstrated that the NF-κB genetic mutation could cause kidney injury and kidney disease progression. However, the association of a gene polymorphism in the transcription factor binding site of NF-κB with kidney disease is not clear.

**Methods:**

We used the Taiwan Biobank database, the University of California, Santa Cruz, reference genome, and a chromatin immunoprecipitation sequencing database to find single nucleotide polymorphisms (SNPs) at potential binding sites of NF-κB. In addition, we performed a case–control study and genotyped 847 patients with ESRD and 846 healthy controls at Tri-Service General Hospital from 2015 to 2016. Furthermore, we used the ChIP assay to identify the binding activity of different genotypes and used Luciferase reporter assay to examine the function of the rs9395890 polymorphism.

**Result:**

The results of biometric screening in the databases revealed 15 SNPs with the potential binding site of NF-κB. Genotype distributions of rs9395890 were significantly different in ESRD cases and healthy controls (*P* = 0.049). The ChIP assay revealed an approximately 1.49-fold enrichment of NF-κB of the variant type TT when compared to that of the wild-type GG in rs9395890 (*P* = 0.027; TT = 3.20 ± 0.16, GT = 2.81 ± 0.20, GG = 1.71 ± 0.18). The luciferase reporter assay showed that the NF-κB binding site activity in T allele was slightly higher than that in G allele, though it is not significant.

**Conclusions:**

Our findings indicate that rs9395890 is associated with susceptibility to ESRD in Taiwan population.

**Electronic supplementary material:**

The online version of this article (10.1186/s12882-019-1471-2) contains supplementary material, which is available to authorized users.

## Background

According to the United States Renal Data System annual report, the incidence and prevalence of end-stage renal disease (ESRD) in Taiwan are among the highest in the world [1, 2]. Taiwan has a 9.8% prevalence of chronic kidney disease (CKD) [3]. The incidence of ESRD in Taiwanese individuals is 450 per million people [2, 4]. CKD-related and ESRD-related costs in Taiwan are US$25,576 per patient-year [5]. CKD development and progression to ESRD involve complex interactions between multiple genetic and environmental factors [6]. Chronic inflammation is an important component of CKD and ESRD. It has a unique role in their pathophysiology and contributes to cardiovascular and all-cause mortality, as well as the development of protein-energy wasting [7, 8]. Genetic factors are important risk factors in the pathogenesis of CKD. The heritability of ESRD is 31.1% in the Taiwanese population [9].

Nuclear factor-kappa B (NF-κB) is an important transcription factor in inflammation and promotes the expression of genes involved in inflammation, such as cytokines and adhesion molecules. NF-κB comprises a family of dimeric transcription factors that regulate the expression of numerous genes involved in inflammation and cell proliferation [10].

NF-κB pathways are activated after a potent stimulus from members of the interleukin-1 and tumor necrosis factor superfamilies or lipopolysaccharides, which rapidly degrade IκB within minutes. Degradation of IκB releases NF-κB. After NF-κB is activated, it moves into the nucleus and induces transcription and expression of specific genes, resulting in inflammation, apoptosis, cell proliferation and differentiation, possibly leading to CKD [11–18].

Several studies have shown that inhibitors of NF-κB activation can regulate the inflammatory response of glomerular mesangial cells [19]. The pathogenesis of glomerular mesangial cell inflammation in patients with kidney disease has been associated with NF-κB activation [20]. A recent study showed that when patients with kidney disease have proteinuria, the NF-κB inflammatory reaction and expression of proinflammatory genes are accelerated [21–23].

Some genetic studies have shown the association of NF-κB genetic mutations with kidney failure and kidney disease progression [10, 24, 25]. NF-κB is an important transcription factor in inflammation. The polymorphisms in NF-κB transcription binding sites have yet to be identified. Therefore, we used bioinformatics technology and the Taiwan Biobank and chromatin immunoprecipitation sequencing (ChIP-Seq) databases to find NF-κB transcription binding site polymorphisms in a Han population. We then performed a case–control study to investigate the association between the polymorphisms and ESRD.

## Methods

### Bioinformatics analysis in the screening of gene processes

**Fig. 1:**
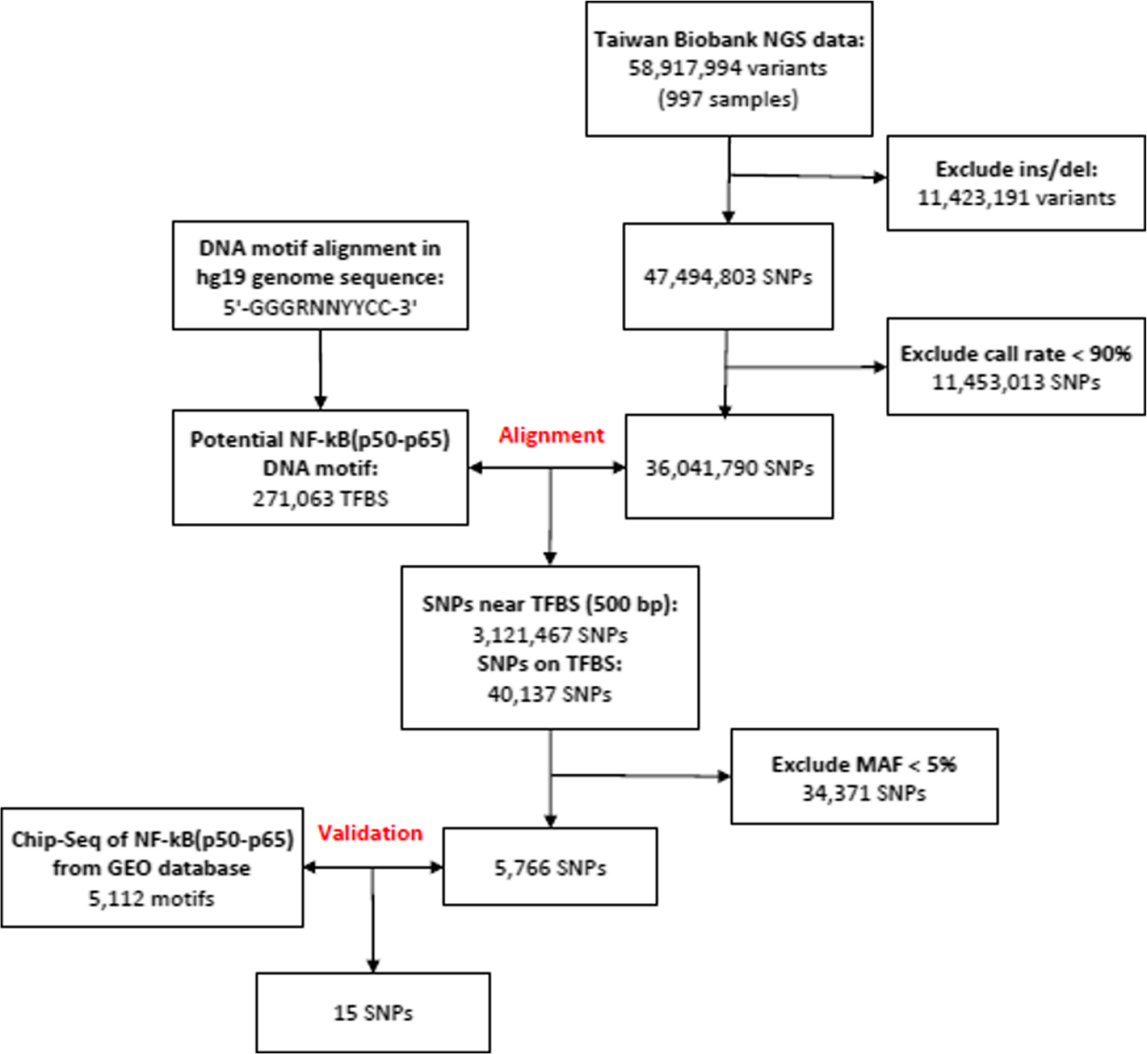
The candidate SNPs screening process by biometrics for this study. First, we used a total of 58,917,994 SNPs (997 samples) in the Taiwan Biobank database to screen for a Taiwanese-specific genetic variation. Then, through genetic alignment of GRCh37/hg19 from the NCBI, we found that NF-κB (p50–p65) contained 271,063 potentials in the human genome based on the sequence of the above binding sites. Of the TFBSs, we compared the remaining 36,041,790 SNPs in the first step and found that there were 3,121,467 SNPs around the 271,063 potential TFBSs, of which 40,137 SNPs were even on the TFBS of NF-κB. Finally, we validated these with the results of the second stage through the ChIP-Seq database to further confirm that these mutations do have a combination of these positions. The 15 SNPs variation may affect NF-κB binding activity

We performed a three-step process for screening of genes (Fig. 1).

#### Screening of genetic variation in a Taiwanese population through a quality control program

First, we used the single nucleotide polymorphism (SNP) database from the Taiwan Biobank. This database includes 58,917,994 SNPs and 997 next-generation sequencing (NGS) samples. Then, we used a human reference genome downloaded from the University of California, Santa Cruz (UCSC; GRCh37/hg19), and all SNPs within 500 kb upstream and downstream of each candidate SNP from the UCSC genome browser (https://genome.ucsc.edu/). We deleted structural variants (insertion/deletion, deletion) because there was no way to use the multifunctional mass spectrometer (mass array) for genotyping. We kept the remaining variants for study and deleted variants with a call rate of less than 90% at the position. Finally, the remaining SNPs were used for further alignment.

#### Sequence alignment techniques using bioinformatics analysis of genetic variations that may affect NF-κB binding

Second, we analyzed genetic variants that may affect NF-κB binding by using bioinformatics sequence alignment techniques and identified the variants located in the transcription factor binding site (TFBS). Prior studies have confirmed that the structure of NF-κB is a dimer consisting of five different related structural proteins: p50, p52, p65 (RelA), RelB, and c-Rel. The combination of the p50 protein and the p65 protein is found [26–28] in almost all cells; as a result, this study explored only the heterodimer of p50/p65. In the past, the TFBS sequence of the identified transcription factor was 5′-GGGRNNYYCC-3′ (R = A or G; N = A, C, G, or T; Y = C or T). We aligned this motif in all 36,041,790 SNPs and in its nearby sequences within 500 kb that included this motif and found 40,137 SNPs that may affect the binding activity.

#### Confirmation by ChIP-Seq that these mutations bind to these positions

Third, we further confirmed that these variations do combine with these locations through ChIP-Seq. After the above screening, we used the method of a previous study on the human genome on the NF-κB ChIP-Seq for analysis of the results of further screening [29]. The study was performed using B cells for ChIP-Seq analysis and analysis of the NF-κB five structural proteins of TFBS and was published in the online Gene Expression Omnibus (GEO) database (GSE55105). We extracted the results of the p50–p65 dimer follow-up screening and found a total of 5112 sequences with alignment to 15 SNPs.

### Study subjects

Next, we conducted a case–control study to identify SNPs related to the NF-κB binding site associated with ESRD. In this study, we collected blood samples and social demographic data from patients admitted to the Tri-Service General Hospital in Taipei, Taiwan, between 2015 and 2016 and then performed real-time polymerase chain reaction (PCR) and genotyping.

We collected data from 847 hemodialysis (HD) patients (male, 50.8%; female, 49.2%; age, 71.84 ± 12.93) from the Tri-Service General Hospital in Taipei, Taiwan. CKD was defined according to the Kidney Disease Outcomes Quality Initiative definitions, and the estimated glomerular filtration rate (eGFR) was calculated using the Modification of Diet in Renal Disease study equation [30, 31]. Study patients were defined as having an eGFR ≤15 mL/min/1.73 m^2^ with clinical signs of uremic syndrome requiring HD. All patients were over 20 years old and had been on HD for more than 6 months. Patients were excluded if they had autoimmune disease, malignancy, or acute or chronic infection. Demographic data of the HD patients included age, sex, diabetes, HD duration, hypertension, education level, and blood biochemical values (white and red blood cells, hemoglobin, blood urea nitrogen [BUN], creatinine, albumin, proteinuria, blood sugar AC, triglycerides, cholesterol, sodium, potassium, calcium, phosphorus, and eGFR). The 846 healthy controls (male, 44.6%; female, 55.4%; age, 73.50 ± 7.21) had no history of renal disease, and their eGFR was ≥60 mL/min/1.73 m^2^. The control group was composed from those undergoing a physical examination at Tri-Service General Hospital. The healthy controls had no microalbuminuria, proteinuria, or hematuria and had normal abdominal/renal ultrasonography findings.

### Ethical statement

The study was reviewed and approved by the institutional ethics committee of the Tri-Service General Hospital (TSGH-1-104-05-006, TSGH-2-106-05-127). After full explanation of the study, written informed consent was obtained from all participants. All clinical and biological samples were collected, and DNA was genotyped following patient consent.

### Genomic DNA extraction and genotyping

The blood samples were extracted from the laboratory by phenol chloroform and stored in a − 20 °C refrigerator for subsequent genotyping and experimental use. Genomic DNA used standard procedures for proteinase K (Invitrogen, Carlsbad, CA, USA) digestion and phenol/chloroform [32] peripheral blood sample separation; then, the samples were genotyped by iPLEX Gold SNP [33]. We assessed the genotyping experiment quality by intrareplication validation. The concordance rate of interreplication validation of 78 samples (approximately 5%) was 100%. Secondary genotyping was performed on 10 random blood samples with PCR according to a previously described protocol for intrareplication validation [34]. After genotyping replication was conducted twice, the concordance rate was 100% between the two genotyping methods.

### Chromatin immunoprecipitation assay and qPCR

We included 9 ESRD patients, GG (*n* = 3), GT (*n* = 3), and TT (*n* = 3), for the ChIP assay. ChIP assays were conducted by using the ChIP Kit ab500 (*Abcam, USA*) and NF-κB antibody (*Proteintech*) according to the manufacturer’s instructions. The immunoprecipitate was eluted with 100 μl DNA purifying slurry, and 2 μl of DNA was used in qPCR. Input DNA and NF-κB-enriched DNA fragments were amplified by using qPCR in a 7500 Fast Real-Time PCR System (*Applied Biosystems*) with primers 5′-ATTCTCACCATGGGAATGG-3′ and 5’GAGGACAGCAAGGTAATAG-3′. The results are shown as percentage input.

### Transient transfection and luciferase assay

NF-κB binding site SNP rs9395890 reporter (from 53820675 to 53821295, 620 bp) was amplified by polymerase chain reaction from one home-made genomic DNA library with the primer pair: 5′: 5′-GGGGTACCGCATCTACGTTCTTAAATGGCC-3′ and 3′: 5′-GGAAGATCTCCTACAGAACCATTACACTCTC-3′ and subcloned into a pGL3 basal reporter (Promega, USA) cut at KpnI and BglII sites. After the sequence verification, we further changed the current T allele into G allele using the QuickChange Lightening Site-directed mutagenesis kit (Agilent Technology). HEK293 cells were grown in Dulbecco’s modified Eagle’s medium supplemented with 10% charcoal/dextran-treated fetal bovine serum. The cells in each well (24-well plate) were transfected with total 1 μg DNA and jetPEI (PolyPlus-transfection, Illkirch, France) according to the manufacturer’s protocol. Luciferase activity was assessed after 24 h post transfection using the Promega Luciferase Assay kit and expressed as mean relative light units (RLU) of two transfected sets. Results shown are representative of at least three independent experiments.

### mRNA expression

We assessed the correlation between genetic variants and mRNA expression of the corresponding genes. Expression quantitative trait loci (eQTL) analysis was also performed using data from the GTEx portal database (https://www.gtexportal.org/home/) and the HapMap Project by a general linear regression model in an additive genetic model [35].

### Statistical analysis

Statistical analysis was performed with R software, version 3.3.1 (R Project for Statistical Computing, Vienna, Austria). Demographic and clinical data between the groups were compared with Student’s *t*-test, and the results for continuous variables were given as the mean ± SD. The allele and genotype frequencies between the different groups were compared with the χ^2^ test when appropriate. The results of ChIP assay qPCR cycles were compared with *ANOVA*. The genetic polymorphism of ESRD risk was calculated using dominant/recessive models. The odds ratios (ORs) and corresponding 95% confidence intervals (CIs) for assessing the effect of the genotype distribution, allele frequencies and binding site activity on ESRD were calculated by logistic regression analysis with adjustment for relevant significant variables. Statistical significance was defined at the 95% level (*P* < 0.05).

## Results

### Screening of genes

#### Next-generation sequencing

We screened for genetic variations in 997 samples from the NGS database in the Taiwan Biobank to determine the total number (58,917,994) of genetic variants in Taiwanese genomes: 11,423,191 were structural variants (insertion/deletion, deletion). There was no way to use the multifunctional mass spectrometer (mass array) for subsequent analysis, and thus we kept only the remaining variants for further study. Therefore, a total of 47,494,803 SNPs were analyzed in detail. Following a quality control program that involved deleting variants with a call rate of less than 90% at the position, 36,041,790 SNPs remained; we then performed sequence alignment analysis.

#### National Center for biotechnology information

We downloaded the human reference gene sequence of GRCh37/hg19 from the National Center for Biotechnology Information (NCBI) in combination with the human biological database in Taiwan and found that NF-κB (p50–p65) contained 271,063 potential variants in the human genome based on the sequence of the above binding sites. Of the TFBSs, we compared the remaining 36,041,790 SNPs in the first step and found that 3,121,467 SNPs were near the 271,063 potential TFBSs, of which 40,137 SNPs were even in the TFBS of NF-κB. Additionally, mutation of this site will likely result in NF-κB (p50–p65) being unable to bind. Finally, a total of 5766 SNPs with a minor allele frequency > 5% were screened for further follow-up by ChIP-Seq analysis due to the limited number of samples subject to subsequent analysis in this study [36].

#### Gene expression omnibus

In the GEO database, there were 5112 positions in the TFBS associated with the p50–p65 dimer. After validating these results with the results of the second stage, the remaining 15 SNPs are shown in Table 1. For SNPs near the DNA sequence, the SNP position as the center ±9 base pairs (the bold font indicates NF-κB) was the expected TFBS. The 15 SNP variations may affect NF-κB binding activity. Finally, we used 15 SNPs obtained from the bioinformatics technology results and the ChIP-Seq database to confirm the relationship with ESRD in this study.

**Table 1:** The ChIP-Seq database found that these SNPs may affect NF-κB binding ability

Chromosome: Location	SNPs	Call rate	MAF	DNA sequence near the SNP
1:11229433	rs17036427	100.0%	8.07% (G → C)	AAAGGCAGG[G/C]ATTTTCCCC
1:19372561	rs79143300	99.7%	8.20% (G → T)	CAATGTGGC[G/T]GGAATTTCC
2:203037846	rs76552560	99.5%	13.46% (G → A)	AAGTCCCCC[G/A]GGAAGTCCC
3:14693650	rs7651075	99.7%	49.40% (G → A)	CCGTGGGTT[G/A]GGAAACTCC
6:44032378	rs59118205	99.7%	5.94% (C → T)	GGGGTTTCC[C/T]CACCATGAT
6:53820994	rs9395890	99.9%	41.57% (T → G)	GTGACAGCT[T/G]GGAAGTCCC
11:30344883	rs11826681	91.6%	41.57% (C → G)	CGTGAGGGG[C/G]ATTTCCAGC
11:85506994	rs11234413	100.0%	9.73% (G → A)	CATCACCAG[G/A]GGAATCTCC
11:94465585	rs2851583	99.9%	5.72% (A → G)	TTCTGAAGG[A/G]AAGTCCCTC
16:1275896	rs9925427	99.3%	12.58% (A → G)	GCAGCGCCC[A/G]GGACTTTCC
16:81444782	rs78229468	95.5%	8.61% (A → G)	TGCTGCTGG[A/G]AAGTTCCTG
16:86553836	rs77836284	99.9%	8.58% (C → T)	GGGGATTTC[C/T]CGCTCGGCT
17:34219824	rs3826454	99.8%	10.50% (A → T)	CCCTTGGGG[A/T]ATTTCCTCA
19:54398240	rs67087171	100.0%	11.53% (G → A)	TAGAAGGGC[G/A]GGATTTCCC
22:29613441	rs7284245	99.7%	16.50% (G → T)	CTTGGGCCG[G/T]GGACTTCCC

### Demographic characteristics

The characteristics of the 846 ESRD and 847 control group subjects are presented in Table 2. The causes of ESRD were diabetes mellitus (DM) in 215 patients (25%), hypertensive nephropathy in 164 (19%), systemic nephropathy in 252 (29%), and other and unknown causes in 136 (16%). There was no significant difference in body mass index. Significant differences in sex, age, DM, hypertension, BUN, serum creatinine, GFR, blood sugar AC, total cholesterol, and triglycerides were observed between patients with ESRD and controls (*P* < 0.001).

**Table 2:** Characteristics of ESRD patients and control subjects

Dependent Independent	Control^a^ (*n* = 847)	ESRD^b^ (*n* = 847)	*p*-value
Male (%)	377 (44.6%)	430 (50.8%)	0.011*
Age (mean ± SD, year)	73.50 ± 7.21	71.84 ± 12.93	0.001*
Diabetes mellitus (%)	105 (12.5%)	372 (80.3%)	< 0.001*
Hypertension (%)	358 (42.7%)	222 (81.3%)	< 0.001*
BMI^c^ (mean ± SD, kg/m^2)^	24.22 ± 3.37	24.66 ± 4.80	0.420
Blood biochemical value (mean ± SD)			
BUN^d^ (mg/dl)	15.90 ± 3.89	73.88 ± 24.72	< 0.001*
Creatinine (mg/dl)	0.83 ± 0.72	9.42 ± 2.78	< 0.001*
Blood sugar PC (mg/dl)	102.48 ± 25.20	148.90 ± 75.40	< 0.001*
Triglycerides (mg/dl)	103.62 ± 40.82	157.02 ± 98.75	< 0.001*
Cholesterol (mg/dl)	185.05 ± 33.35	162.42 ± 45.16	< 0.001*
Glomerular filtration rate (GFR) (mL/min/1.73 m^2^)	93.81 ± 23.76	5.73 ± 2.45	< 0.001*

*p < 0.05

^a^: Control: GFR > 60; ^b^: Case: Hemodialysis patients, GFR < 15; ^c^: Body Mass Index; ^d^: Blood urea nitrogen

### Association analyses of NF-κB binding site gene polymorphisms with susceptibility to ESRD

In the gene screening process, the call rate of all 15 SNPs was > 90%, and the genotypes of these SNPs were in Hardy–Weinberg equilibrium (*P* > 0.05). When we calculated our sample size, the power was > 50% and the OR was set at 1.5 to detect the real effects of expected NF-κB binding site SNPs. Two SNPs were nonfrequency SNPs under the allele model (rs2851583, rs76552560), and a suitable primer could not be found for three SNPs (rs11234413, rs3826454, rs67087171). Finally, genotyping results were obtained for 10 SNPs. Our results showed that SNP rs9395890 had a significant association with ESRD risk according to genotype (*P* = 0.041; Table 3).

**Table 3:** Genotype distribution of NF-κB binding site SNPs with ESRD cases and control group

SNPs	Control (*N* = 847)	ESRD (*N* = 847)	Crude-OR (95% CI)	*p*-value	Adj-OR (95% CI) ^a^	*p*-value
rs11826681C/G				0.385		0.251
CC	304 (35.9%)	288 (34.3%)	1.00		1.00	
CG	421 (49.7%)	418 (48.9%)	1.03 (0.83–1.27)	0.798	1.00 (0.81–1.25)	0.970
GG	122 (14.4%)	141 (16.8%)	1.22 (0.91–1.63)	0.181	1.26 (0.94–1.70)	0.128
rs17036427G/C				0.825		0.826
GG	723 (85.4%)	718 (84.8%)	1.00		1.00	
GC	119 (14.0%)	122 (14.4%)	1.03 (0.79–1.36)	0.819	1.00 (0.75–1.32)	0.990
CC	5 (0.6%)	7 (0.8%)	1.41 (0.45–4.46)	0.559	1.44 (0.45–4.63)	
rs59118205C/T				0.624		0.905
CC	745 (88.0%)	753 (89.0%)	1.00		1.00	
TC	98 (11.6%)	92 (10.8%)	0.92 (0.68–1.24)	0.583	0.93 (0.68–1.27)	0.935
TT	4 (0.5%)	2 (0.2%)	0.49 (0.09–2.71)	0.417	1.09 (0.15–7.87)	0.662
rs7284245G/T				0.234		0.220
GG	611 (72.3%)	582 (69.5%)	1.00		1.00	
GT	211 (25.0%)	247 (28.4%)	1.18 (0.95–1.47)	0.127	1.15 (0.92–1.44)	0.210
TT	23 (2.7%)	18 (2.1%)	0.82 (0.44–1.54)	0.539	0.70 (0.36–1.34)	0.278
rs7651075G/A				0.104		0.139
GG	219 (25.9%)	185 (22.0%)	1.00		1.00	
AG	398 (46.9%)	440 (51.5%)	1.29 (1.02–1.64)	0.036	1.26 (0.99–1.61)	0.062
AA	230 (27.2%)	222 (26.4%)	1.14 (0.87–1.50)	0.331	0.12 (0.85–1.48)	0.417
rs77836284C/T				0.775		0.662
CC	703 (83.8%)	709 (84.4%)	1.00		1.00	
TC	133 (14.9%)	130 (14.6%)	0.98 (0.74–1.28)	0.858	1.03 (0.78–1.36)	0.856
TT	11 (1.3%)	8 (1.0%)	0.72 (0.29–1.80)	0.484	0.65 (0.25–1.70)	0.378
rs78229468A/G				0.518		0.719
AA	697 (82.4%)	696 (82.3%)	1.00		1.00	
GA	146 (17.1%)	143 (16.8%)	0.98 (0.76–1.26)	0.881	0.98 (0.75–1.27)	0.859
GG	4 (0.5%)	8 (0.9%)	2.00 (0.60–6.68)	0.258	1.65 (0.47–5.74)	0.432
rs79143300G/T				0.668		0.464
GG	737 (87.0%)	731 (86.3%)	1.00		1.00	
GT	105 (12.4%)	113 (13.3%)	1.09 (0.82–1.44)	0.574	1.11 (0.83–1.49)	0.471
TT	5 (0.6%)	3 (0.4%)	0.60 (0.14–2.54)	0.492	0.43 (0.08–2.26)	0.321
rs9395890G/T				0.031*		0.049*
GG	153 (18.1%)	138 (16.4%)	1.00		1.00	
GT	419 (49.5%)	379 (45.1%)	1.00 (0.77 to 1.31)	0.983	0.98 (0.75 to 1.29)	0.110
TT	274 (32.4%)	324 (38.5%)	1.31 (0.99 to 1.74)	0.059	1.26 (0.95 to 1.68)	0.908
rs9925427A/G				0.902		0.826
AA	638 (77.2%)	665 (79.1%)	1.00		1.00	
GA	208 (22.7%)	192 (20.9%)	0.95 (0.75–1.20)	0.650	0.93 (0.73–1.18)	0.537
GG	1	0	0.00 (0.00 - inf)	0.969	0.00 (0.00-inf)	0.969

*p < 0.05

^a^: adjust: gender, age, BMI, Hypertension, DM

### Allele frequencies for the NF-κB binding site gene polymorphisms with susceptibility to ESRD

There was a significant association (*P* = 0.049; Table 3) between rs9395890 and ESRD. The SNP rs9395890 with the T allele is associated with ESRD risk in the allele model (*P* = 0.013; odds ratio [OR] = 1.31, 95% confidence interval [CI] = 1.06 to 1.62; Table 4) and recessive model (*P* = 0.008; odds ratio [OR] = 1.31, 95% confidence interval [CI] = 1.07 to 1.60; Table 4). There were no significant differences in genotype or allele frequencies in the other nine SNPs between patients with ESRD and controls (Additional file 1).

**Table 4:** The allele frequency of rs9395890 for the NF-κB binding site SNPs with ESRD cases and control group

SNPs	Control (*N* = 847)	ESRD (*N* = 847)	Crude-OR (95% CI)	*p*-value	Adj-OR (95% CI) ^a^	*p*-value
rs9395890						
Allele model				0.008*		0.013*
G	725 (43%)	655 (39%)	1.00		1.00	
T	967 (57%)	1027 (61%)	1.31 (1.07 to 1.60)		1.31 (1.06 to 1.62)	
Dominant model				0.362		0.983
G	153 (18.1%)	138 (16.4%)	1.00		1.00	
GT + T	693 (81.9%)	703 (83.6%)	1.12 (0.87 to 1.45)	0.362	1.00 (0.77 to 1.31)	0.983
Recessive model				0.008*		0.008*
G + GT	572 (67.6%)	517 (61.5%)	1.00		1.00	
T	274 (32.4%)	324 (38.5%)	1.31 (1.07 to 1.60)	0.008*	1.31 (1.07 to 1.60)	0.008*

*p < 0.05

^a^: adjust: gender, age, BMI, Hypertension, DM

### ChIP assay identified NF-κB binding site rs9395890 enrichment

**Fig. 2:**
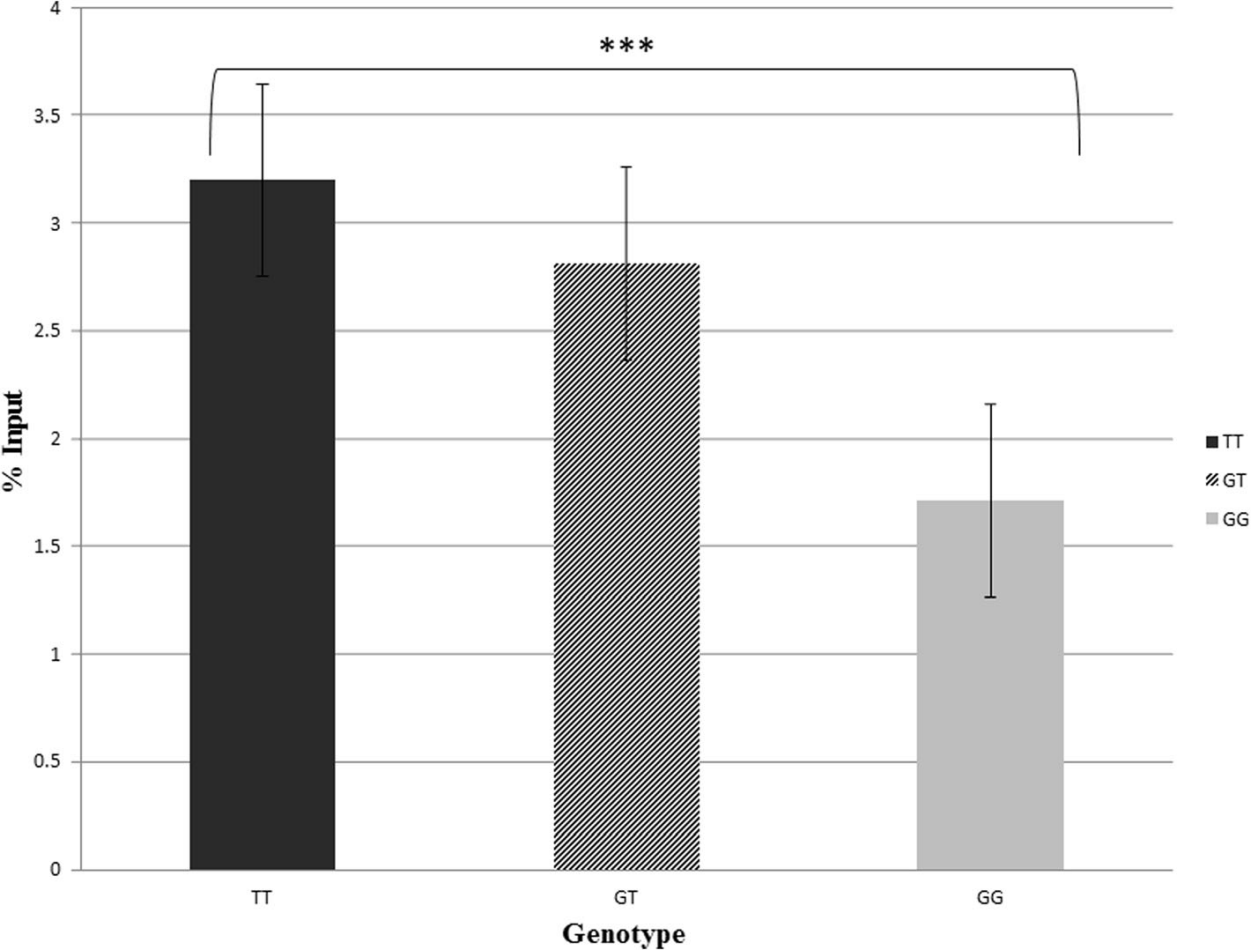
Confirmation of NF-κB binding ability in the rs9395890 by using ChIP assay. The representative data of enrichment of NF-κB of three genotypes, GG (*n* = 3), GT (*n* = 3), and TT (*n* = 3), in the rs9395890 by using chromatin immunoprecipitation assay (ChIP). Real-time qPCR was performed to measure the amount of with or without NF-κB enriched fragments. The ChIP-assay reveals that around 1.49 times enrichment of NF-κB of the variant type TT when compared to that of the wild type GG in the rs9395890. The SNP at NF-KB transcription binding site rs9395890 have high binding ability in TT type than GG type. The results are shown in % input (ChIP/input). The mean ± SEM is given for each construct from three experiments (*P* = 0.027; TT = 3.20 ± 0.16, GT = 2.81 ± 0.20, GG = 1.71 ± 0.18) (Table 5)

We included 9 ESRD patients, GG (*n* = 3), GT (*n* = 3), TT (*n* = 3), in the ChIP assay experiments. Real-time qPCR was performed to measure the amount of NF-κB-enriched DNA fragments. The ChIP assay revealed an approximately 1.49-fold enrichment of NF-κB of the variant type TT when compared to that of the wild-type GG in rs9395890 (*P* = 0.027; TT = 3.20 ± 0.16, GT = 2.81 ± 0.20, GG = 1.71 ± 0.18). When the SNP rs9395890 was type TT, the NF-κB transcription binding activity was higher than that of the GG type (TT: *P* < 0.001; odds ratio [OR] = 1.50, 95% confidence interval [CI] = 1.20–1.77; GT: *P* < 0.001; odds ratio [OR] = 1.12, 95% confidence interval [CI] = 1.01–1.38; Table 5, Fig. 2).

**Table 5:** The ChIP-assay reporter that the ability of SNP rs9395890 in different genotype at NF-KB binding site

Independent variable	Ct	*p*-value	OR (95% CI)	*p*-value
Genotype		0.027*		< 0.001*
GG	1.71 ± 0.18		1.00	
GT	2.81 ± 0.20		1.12 (1.01 to 1.38)	< 0.001*
TT	3.20 ± 0.16		1.50 (1.20 to 1.77)	< 0.001*

*Ct* mean ± SEM

*p < 0.05

### Comparison of NF-κB binding activity of T and G allele of the rs9395890 T/G

**Fig. 3:**
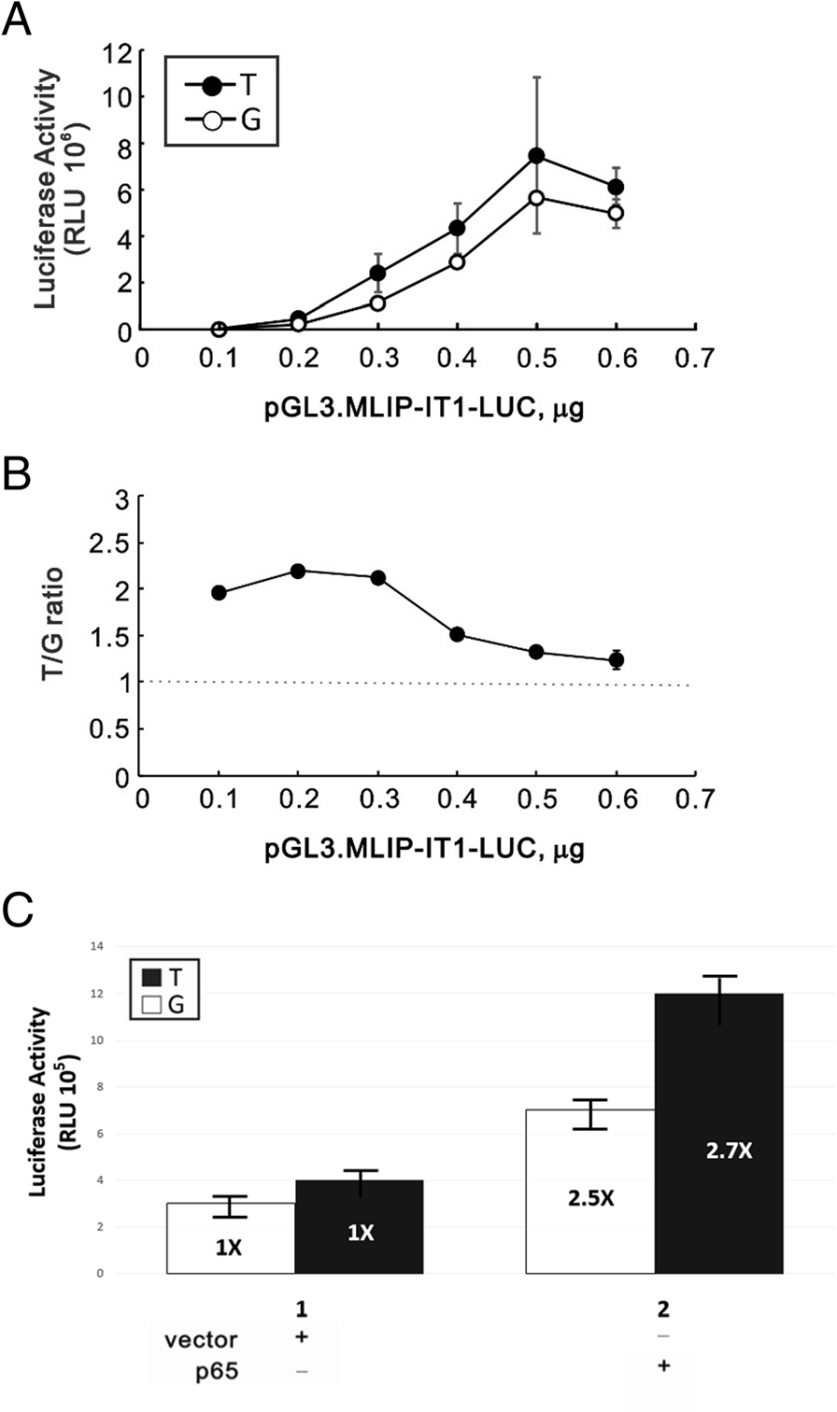
The effect of T/G allele within the NF-κB site on the MLIP-IT1 reporter activity. **a** HEK293 cells were transiently transfected indicated amount of pGL3.MLIP-IT1-LUC containing T or G allele and **b** the plot of T/G ratio. Dotted line represent the baseline of one fold. **c** the Luciferase activity upon overexpression of P65 or not in T and G allele, respectively. These data are the averages of three experiments (mean ± S.D.; *n* = 3)

To establish whether the SNP rs9395890 were functional. We investigated influenced NF-κB binding site activity using a luciferase reporter assay in HEK293 cells. The results are shown in Fig. 3. First, we examined the background of reporter activity in T and G allele by a dose dependent of the amount of pGL3.MLIP-IT1-LUC. It showed that the background activity were higher upon the increasing amount of pGL3.MLIP-IT1-LUC, however, there were no difference in that between T and G allele (Fig. 3a). Further, the functionality of T allele and G allele were observed by upon overexpression of p65 or not in the reporter assay, respectively. Data showed that the luciferase activity in T allele (2.7X) was slightly higher than that in G allele (2.5X), though there are no significant difference (*P* = 0.589).

### The SNP rs9395890 in silico functional validation

**Fig. 4:**
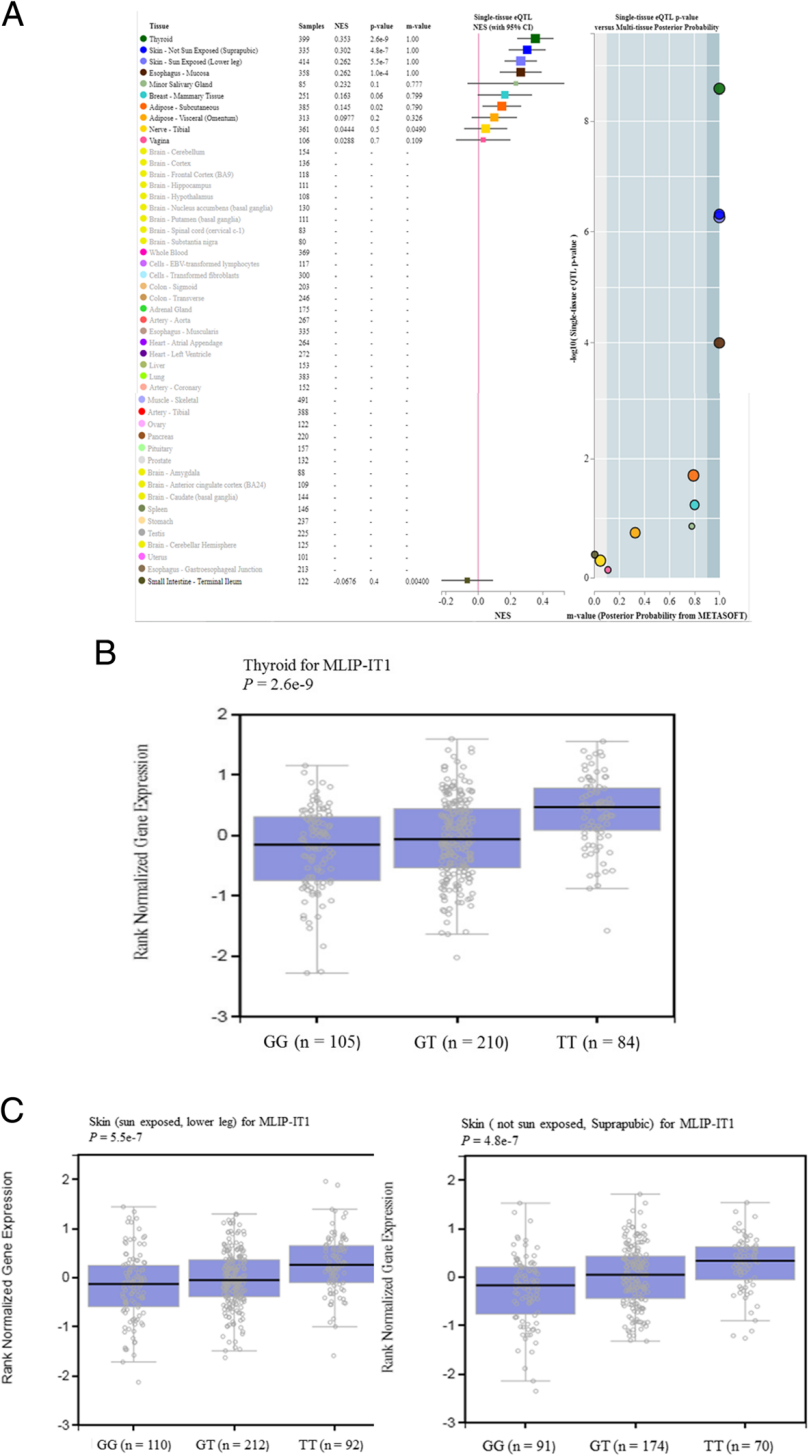
The result of expression quantitative trait loci analysis (eQTL) from the GETx-Portal (https://www.gtexportal.org/home/) for MLIP-IT1 rs9395890 in (**a**) The multi-tissue eQTL Plot about Thyroid (*P* = 2.6*10^− 9^), Skin - Not Sun Exposed (Suprapubic) (*P* = 4.8*10^− 7^), Skin - Sun Exposed (Lower leg) (*P* = 5.5*10^− 7^), Esophagus – Mucosa (*P* = 1.0*10^− 4^), Minor Salivary Gland (*P* = 0.1), Breast - Mammary Tissue (*P* = 0.06), Adipose - Subcutaneous (*P* = 0.02), Adipose - Visceral (Omentum) (*P* = 0.2), Nerve - Tibial (*P* = 0.5), Vagina (*P* = 0.7), Small Intestine - Terminal Ileum (*P* = 0.4); (**b**)(**c**) The gene expression box plot by QTL analysis using HapMap data for MLIP-IT1 rs9395890 with thyroid and skin tissue [35]

When we mapped SNPs using the NCBI database (https://www.ncbi.nlm.nih.gov/projects/SNP/snp_ref.cgi?rs=9395890), rs9395890 was located in intron, chromosome 6, LOC101927189, and the nearest downstream gene was MLIP-IT1. We further conducted MLIP-IT1 mRNA expression quantitative trait loci (eQTL) analysis by searching the GTEx portal (https://www.gtexportal.org/home/). We found that for MLIP-IT1, the T allele was significantly associated with increased expression levels in the following categories: Thyroid (*P* = 2.6*10^− 9^; Fig. 4b), Skin - Not Sun Exposed (Suprapubic) (*P* = 4.8*10^− 7^; Fig. 4c), Skin - Sun Exposed (Lower leg) (*P* = 5.5*10^− 7^; Fig. 4c), Esophagus - Mucosa (*P* = 1.0*10^− 4^; Fig. 4a), Minor Salivary Gland (*P* = 0.1; Fig. 3a), Breast - Mammary Tissue (*P* = 0.06; Fig. 4a), Adipose - Subcutaneous (*P* = 0.02; Fig. 4a), Adipose - Visceral (Omentum) (*P* = 0.2; Fig. 4a), Nerve - Tibial (*P* = 0.5; Fig. 4a), Vagina (*P* = 0.7; Fig. 4a), and Small Intestine - Terminal Ileum (*P* = 0.4; Fig. 4a).

## Discussion

Our results suggested that there is a significant correlation between rs9395890 and ESRD risk. This genetic association study employed bioinformatics technology and epidemiological approaches that make it different from other studies. Previous reports included more genetic and molecular epidemiological studies of ESRD in genome-wide association studies (GWAS). GWAS can explore the etiological contribution of genetic variants throughout the whole genome without applying previously hypothesis. However there are very few detected causal variants [37]. Therefore, we provided an approach to use a hybrid method consisting of candidate gene and epidemiologic approaches.

The research of inflammatory transcription factor (NF-κB) associated SNPs has been investigated in a few previous studies [38–41]. However, we addressed the importance of genetic polymorphisms in determining ESRD in this study. We were able to identify loci and information about which genes were associated with complex diseases [42, 43]. In our study, we used methodological approach that combined the NGS, NCBI, and GEO online databases to find target SNPs and used epidemiological methods to confirm the findings in a case–control study. A previous study in 2014 also used publicly available genomic data and bioinformatics platforms to provide additional evidence for the TFBSs of SNPs of the ERα-regulating sequence at 21q22.3, which are important in determining breast cancer progression [43].

Immune and inflammatory factors have important roles in the pathogenesis of kidney diseases [44, 45]. Based on previous studies, the transcription factor NF-κB regulates the expression of various genes that have an important role in the regulation of immunity and inflammation in disease [10]. NF-κB regulates T cells, particularly the T helper 17 cells, which mainly affect the pathogenesis of autoimmunity and inflammation [46]. Several studies have shown the cell-intrinsic role of NF-κB in T cell generation [47, 48]. In the NF-κB pathway, when cells are unstimulated, NF-κB is bound to I*k*B*a* and I*k*B*b* in the cytoplasm, which prevents NF-κB from entering the nucleus [49]. When these cells are stimulated, specific kinases phosphorylate I*k*B, allowing degradation by proteasomes [50, 51]. The NF-κB released from I*k*B results in the passage of NF-κB into the nucleus, and NF-κB binds to target sequences in the promoter regions of target genes, leading to the expression of many genes involved in immune and inflammatory responses [52].

The NF-κB signaling pathway regulated renal inflammation and the progression of ESRD. Histological evidence of NF-κB activation has been associated with human renal disease with diabetes, glomerular disease, and acute kidney injury [53]. The NF-κB transcription of multiple proinflammatory molecules, such as cytokines, chemokines, allograft antigens, adhesion molecules, and reactive oxygen, in response to renal injury [54]. The SNPs at NF-κB transcription binding site are functional polymorphisms that might regulatory polymorphisms situated in the noncoding regions of the genes which may affect gene product protein due to the transcriptional alterations [55].

In the past, we knew that the NF-κB transcription factor binding site was involved in the regulation of downstream inflammatory genes, which in turn affected the progression of disease and the deterioration of inflammation. However, the results of this study found an association between ESRD risk and the NF-κB binding site SNP rs9395890. Furthermore, we used a ChIP assay to identify NF-KB binding activity with different genotypes. We found that the NF-KB binding activity at SNP rs9395890 with the TT type was higher than that of the GG type. And we assessed the functionality of the NF-κB binding site rs9395890 T/G polymorphism for effects activity by luciferase reporter assay. Our experimental Fig. 3 showed that the transcriptional activity of the T allele was higher than G allele, but the relative light units (RLU) data was no significant difference between with T and G allele. So far there were no study about the rs9395890 and MLIP-IT1. However it might be the distance between rs9395890 and MLIP-IT1 is too far away.

Furthermore, the results from GTEx portal demonstrated that the T allele was significantly associated with increasing expression levels of rs9395890 in multiple tissues, suggesting that rs9395890 may modulate the risk of ESRD, possibly through a mechanism of modulating gene expression [35].

SNP rs9395890 is an intron variant located on chromosome 6: 53820994 in front of the MLIP-IT1 gene − 42694 bp. MLIP-IT1 is a noncoding RNA gene, and MLIP-IT1 is a responding gene of rs9395890. Noncoding RNA is not translated into protein but causes transcription factor binding protein and expression of downstream genetics. We suspect that a mutation in this site will affect the function of this gene in MLIP-IT1, which increases the risk of ESRD. To our knowledge, few studies have reported MLIP-IT1 and rs9395890 [56]. DNA is transcribed to mRNA by transcription factors, which then initiate their function. Noncoding RNA occurs during DNA transcription to RNA, when a portion of RNA cannot become mRNA. Noncoding RNA regulates gene transcription function and protein transport. More studies have focused on noncoding RNA and its association with chromatin remodeling, gene transcription, protein transport, and trafficking. Noncoding RNA also has important roles in most human diseases, including coronary artery diseases, autoimmune diseases, neurological disorders, and various cancers [37, 43, 57]. Specifically, we found that the rs9395890 T allele was associated with the risk of ESRD. The T allele mRNA expression levels were higher than those of the G allele in thyroid, skin and mucosa inflammation disease according to data from the GTEx portal. These results are consistent with our ChIP assay data (TT binding activity higher than GG; Fig. 2) [35].

However, we did not confirm that the NF-κB transcription binding site SNP rs9395890 and the responding gene MLIP-IT1 regulated the mechanism of ESRD risk. Therefore, an experiment to identify the association between rs9395890 MLIP-IT1 RNA expression and ESRD risk is necessary in the future.

Our study has some limitations. First, to our knowledge, no studies have related SNPs of NF-κB transcription binding sites to disease. Our study used bioinformatics technology, that is, the NGS, NCBI, and GEO online databases, to screen transcription binding site genetics. Furthermore, we used our case–control groups for genotyping to confirm that rs9395890 was associated with ESRD. We used GEO database-involved B cells for ChIP-Seq analysis, but it was difficult to obtain renal cells to repeat verification. Second, the odds ratio of rs9395890 was very low, but this is a limitation of an observational study. Third, our study sample size was not large enough. Bonferroni correction could not be performed. However, only one SNP was significantly correlated in our study, and the results of functional analysis were indeed related to ESRD risk. Our study included both a genetic association test and a functional analysis, and the results were consistent (*p* < 0.05 in both tests). Because of the double statistical test setting, we consider that the type 1 error rate in our setting is less than that in general genetic association studies using Bonferroni correction, and thus the evidence level provided by our study is sufficient even though we cannot conduct Bonferroni correction. In summary, we conclude that SNP rs9395890 plays a key role in the incidence of ESRD.

## Conclusion

Our study demonstrated that SNP rs9395890 might contribute to NF-κB transcription binding site ability and might exert an effect on MLIP-IT1 activity. The function of MLIP-IT1 with regard to ESRD progression risk and survival should be explored further.

## Additional file

Additional file 1: Genotype distributions and allele frequencies for the NF-κB binding site SNPs in ESRD patients and control group. (DOCX 40 kb)

## Data Availability

The data analyzed in this study can be accessed by sending a request to the corresponding author.

## Abbreviations

BUN: Blood urea nitrogen
ChIP-Seq: Chromatin immunoprecipitation sequencing
CIs: Confidence intervals
CKD: Chronic kidney disease
eGFR: Estimated glomerular filtration rate
ESRD: End-stage renal disease
GEO: Gene Expression Omnibus
HD: Hemodialysis
NCBI: National Center for Biotechnology Information
NF-κB: Nuclear factor-kappaB
NGS: Next generation
ORs: Odds ratios
SNP: Single nucleotide polymorphisms
TFBS: Transcription factor binding site
TSGH: Tri-Service General Hospital
UCSC: University of California, Santa Cruz
DM: Diabetes mellitus
GWAS: Genome-wide association studies
