# Supplementary material for: Investigation of the variants at the binding site of inflammatory transcription factor NF-κB in patients with end-stage renal disease
Source: BMC Nephrol. 2019 Aug 5;20:300. doi: 10.1186/s12882-019-1471-2 (PMC6683452; doi:10.1186/s12882-019-1471-2)
Supplement: Supplementary file 1 — Genotype distributions and allele frequencies for the NF-κB binding site SNPs in ESRD patients and control group. (DOCX 40 kb) [file 12882_2019_1471_MOESM1_ESM.docx]

**Supplementary**

S1. Genotype distributions and allele frequencies for the NF-κB binding site SNPs in ESRD patients and control group.

|  | Control  (n=847) | ESRD  (n=847) | Crude OR  (95%CI) | *P-value* | Adjusted OR ^a^  (95%CI) | *P-value* |
| --- | --- | --- | --- | --- | --- | --- |
| rs11826681C/G |  |  |  |  |  |  |
| Genotypes |  |  |  | 0.385 |  | 0.251 |
| CC | 304 (35.9%) | 288 (34.3%) | 1.00 |  | 1.00 |  |
| CG | 421 (49.7%) | 418 (48.9%) | 1.03 (0.83 - 1.27) | 0.798 | 1.00 (0.81 - 1.25) | 0.970 |
| GG | 122 (14.4%) | 141 (16.8%) | 1.22 (0.91 - 1.63) | 0.181 | 1.26 (0.94 - 1.70) | 0.128 |
| Allele model |  |  |  | 0.229 |  | 0.179 |
| C | 1029 (61%) | 986 (59%) | 1.00 |  | 1.00 |  |
| G | 665 (32%) | 692 (41%) | 1.09 (0.95 - 1.25) |  | 1.11 (0.95 - 1.29) |  |
| Dominant model |  |  |  | 0.203 |  | 0.181 |
| G | 122 | 141 | 1.00 |  | 1.00 |  |
| CG+C | 725 | 706 | 0.84 (0.65-1.10) | 0.203 | 0.82 (0.61 - 1.10) | 0.181 |
| Recessive model |  |  |  | 0.415 |  | 0.181 |
| G+CG | 543 | 559 | 1.00 |  | 1.00 |  |
| C | 304 | 288 | 0.92 (0.75 - 1.12) | 0.415 | 0.82 (0.61 - 1.10) | 0.181 |
| **rs17036427G/C**  Genotypes |  |  |  | 0.825 |  | 0.840 |
| GG | 723 (85.4%) | 718 (84.8%) | 1.00 |  | 1.00 |  |
| GC | 119 (14.0%) | 122 (14.4%) | 1.03 (0.79 – 1.36) | 0.819 | 1.00 (0.75 – 1.32) | 0.990 |
| CC | 5 (0.6%) | 7 (0.8%) | 1.41 (0.45 - 4.46) | 0.559 | 1.44 (0.45 - 4.63) |  |
| Allele model |  |  |  | 0.679 |  | 0.713 |
| G | 1565 (92%) | 1558 (92%) | 1.00 |  | 1.00 |  |
| C | 129 (8%) | 136 (8%) | 1.06(0.82-1.37) |  | 1.05 (0.81 – 1.37) |  |
| Dominant model |  |  |  | 0.733 |  | 0.559 |
| G | 723 | 718 | 1.00 |  | 1.00 |  |
| GC+C | 124 | 129 | 1.05 (0.80-1.37) | 0.733 | 1.41 (0.45 - 4.46) | 0.559 |
| Recessive model |  |  |  | 0.564 |  | 0.603 |
| G+GC | 842 | 840 | 1.00 |  | 1.00 |  |
| C | 5 | 7 | 1.40 (0.44-4.44) | 0.564 | 1.37 (0.42 - 4.42) | 0.603 |
| **rs79143300G/T**  Genotypes |  |  |  | 0.668 |  | 0.464 |
| GG | 737 (87.0%) | 731 (86.3%) | 1.00 |  | 1.00 |  |
| GT | 105 (12.4%) | 113 (13.3%) | 1.09 (0.82 – 1.44) | 0.574 | 1.11 (0.83 – 1.49) | 0.471 |
| TT | 5 (0.6%) | 3 (0.4%) | 0.60 (0.14 – 2.54) | 0.492 | 0.43 (0.08 – 2.26) | 0.321 |
| Allele model |  |  |  | 0.083 |  | 0.221 |
| G | 1484 (93%) | 1560 (92%) | 1.00 |  | 1.00 |  |
| T | 108 (7%) | 134 (8%) | 1.26 (0.97 - 1.64) |  | 1.33 (1.01 - 1.75) | 0.043 |
| Dominant model |  |  |  | 0.091 |  |  |
| G | 742 | 718 | 1.00 |  | 1.00 |  |
| GT+T | 105 | 129 | 1.27 (0.96 - 1.68) |  | 1.72 (0.41 - 7.23) | 0.458 |
| Recessive model |  |  |  | 0.726 |  | 0.458 |
| G+GT | 844 | 842 | 1.00 |  | 1.00 |  |
| T | 3 | 5 | 1.67 (0.40 - 7.01) |  | 1.72 (0.41 - 7.23) | 0.458 |
| **rs59118205C/T**  Genotypes |  |  |  | 0.624 |  | 0.905 |
| CC | 745 (88.0%) | 753 (89.0%) | 1.00 |  | 1.00 |  |
| TC | 98 (11.6%) | 92 (10.8%) | 0.92 (0.68 - 1.24) | 0.583 | 0.93 (0.68 – 1.27) | 0.935 |
| TT | 4 (0.5%) | 2 (0.2%) | 0.49 (0.09 - 2.71 ) | 0.417 | 1.09 (0.15 – 7.87) | 0.662 |
| Allele model |  |  |  | 0.429 |  | 0.577 |
| C | 1588 (94%) | 1597 (94%) | 1.00 |  | 1.00 |  |
| T | 106 (6%) | 95 (6%) | 0.89 (0.67-1.19) |  | 0.92 (0.68 – 1.24) |  |
| Dominant model |  |  |  | 0.544 |  | 0.632 |
| C | 745 | 753 | 1.00 |  | 1.00 |  |
| TC+T | 102 | 94 | 0.91(0.68-1.23) | 0.544 | 0.93 (0.69 - 1.26) | 0.632 |
| Recessive model |  |  |  | 0.422 |  | 0.473 |
| C+TC | 843 | 845 | 1.00 |  | 1.00 |  |
| T | 4 | 2 | 0.50 (0.09-2.73) | 0.422 | 0.53 (0.10 - 2.98) | 0.473 |
| **rs7284245G/T**  Genotypes |  |  |  | 0.234 |  | 0.220 |
| GG | 611 (72.3%) | 582 (69.5%) | 1.00 |  | 1.00 |  |
| GT | 211 (25.0%) | 247 (28.4%) | 1.18 (0.95 - 1.47) | 0.127 | 1.15 (0.92 - 1.44) | 0.210 |
| TT | 23 (2.7%) | 18 (2.1%) | 0.82 (0.44 – 1.54) | 0.539 | 0.70 (0.36 – 1.34) | 0.278 |
| Allele model |  |  |  | 0.364 |  | 0.693 |
| G | 1433 (85%) | 1402 (84%) | 1.00 |  | 1.00 |  |
| T | 257 (15%) | 274 (16%) | 1.09(0.91-1.31) |  | 1.04 (0.85 - 1.27) |  |
| Dominant model |  |  |  | 0.123 |  | 0.074 |
| G | 611 | 582 | 1.00 |  | 1.00 |  |
| GT+T | 236 | 265 | 1.18 (0.96-1.45) |  | 1.22 (0.98 - 1.51) | 0.074 |
| Recessive model |  |  |  | 0.430 |  | 0.231 |
| G+GT | 824 | 829 | 1.00 |  | 1.00 |  |
| T | 23 | 18 | 0.78 (0.42-1.45) | 0.430 | 0.67 (0.35 - 1.28) | 0.231 |
| **rs7651075G/A** |  |  |  |  |  |  |
| Genotypes |  |  |  | 0.104 |  | 0.139 |
| GG | 219 (25.9%) | 185 (22.0%) | 1.00 |  | 1.00 |  |
| AG | 398 (46.9%) | 440 (51.5%) | 1.29 (1.02 - 1.64) | 0.036 | 1.26 (0.99 - 1.61) | 0.062 |
| AA | 230 (27.2%) | 222 (26.4%) | 1.14 (0.87 - 1.50) | 0.331 | 0.12 (0.85 - 1.48) | 0.417 |
| Allele model |  |  |  | 0.367 |  | 0.514 |
| G | 835 (49%) | 803 (48%) | 1.00 |  | 1.00 |  |
| A | 857 (51%) | 877 (52%) | 1.06 (0.93-1.22) | 0.331 | 1.05 (0.91 - 1.22) |  |
| Dominant model |  |  |  | 0.053 |  | 0.027 |
| G | 219 | 185 | 1.00 |  | 1.00 |  |
| AG+A | 628 | 662 | 1.25 (1.00-1.56) | 0.053 | 1.31 (1.03 - 1.66) | 0.027 |
| Recessive model |  |  |  | 0.660 |  | 0.245 |
| G+AG | 617 | 625 | 1.00 |  | 1.00 |  |
| A | 230 | 222 | 0.95 (0.77-1.18) | 0.660 | 0.87 (0.69 - 1.10) | 0.245 |
| **rs77836284C/T**  Genotypes |  |  |  | 0.775 |  | 0.662 |
| CC | 703 (83.8%) | 709 (84.4%) | 1.00 |  | 1.00 |  |
| TC | 133 (14.9%) | 130 (14.6%) | 0.98 (0.74 - 1.28) | 0.858 | 1.03 (0.78 - 1.36) | 0.856 |
| TT | 11 (1.3%) | 8 (1.0%) | 0.72 (0.29 - 1.80) | 0.484 | 0.65 (0.25 - 1.70) | 0.378 |
| Allele model |  |  |  | 0.614 |  | 0.761 |
| C | 1531 (91%) | 1541 (92%) | 1.00 |  | 1.00 |  |
| T | 147 (9%) | 139 (8%) | 0.94 (0.74-1.20) |  | 0.96 (0.75 - 1.24) |  |
| Dominant model |  |  |  | 0.696 |  | 0.816 |
| C | 703 | 709 | 1.00 |  | 1.00 |  |
| TC+T | 144 | 138 | 0.95 (0.74-1.23) | 0.696 | 0.97 (0.74 - 1.26) | 0.816 |
| Recessive model |  |  |  | 0.491 |  | 0.539 |
| C+TC | 836 | 839 | 1.00 |  | 1.00 |  |
| T | 11 | 8 | 0.72 (0.29-1.81) | 0.491 | 0.74 (0.29 - 1.91) | 0.539 |
| **rs78229468A/G**  Genotypes |  |  |  | 0.518 |  | 0.719 |
| AA | 697 (82.4%) | 696 (82.3%) | 1.00 |  | 1.00 |  |
| GA | 146 (17.1%) | 143 (16.8%) | 0.98 (0.76 - 1.26) | 0.881 | 0.98 (0.75 - 1.27) | 0.859 |
| GG | 4 (0.5%) | 8 (0.9%) | 2.00 (0.60 – 6.68) | 0.258 | 1.65 (0.47 – 5.74) | 0.432 |
| Allele model |  |  |  | 0.766 |  | 0.767 |
| A | 1539 (91%) | 1534 (91%) | 1.00 |  | 1.00 |  |
| G | 153 (9%) | 158 (9%) | 1.04 (0.82-1.31) |  | 1.04 (0.81 – 1.33) |  |
| Dominant model |  |  |  | 0.949 |  | 0.881 |
| A | 697 | 696 | 1.00 |  | 1.00 |  |
| GA+G | 150 | 151 | 1.01 (0.79-1.29) | 0.949 | 0.98 (0.76 - 1.26) | 0.881 |
| Recessive model |  |  |  | 0.256 |  | 0.252 |
| A+GA | 843 | 839 | 1.00 |  | 1.00 |  |
| G | 4 | 8 | 2.01 (0.60-6.70) | 0.256 | 2.04 (0.60 - 6.93) | 0.252 |
| **rs9395890T/G**  Genotypes |  |  |  | 0.031* |  | 0.041* |
| TT | 274 (32.4%) | 324 (16.4%) | 1.00 |  | 1.00 |  |
| GT | 419 (49.5%) | 379 (45.1%) | 0.76 (0.62 – 0.95) | 0.013* | 0.77 (0.62 – 0.96) | 0.019* |
| GG | 153 (18.1%) | 138 (16.4%) | 0.76 (0.58 – 1.01) | 0.059 | 0.79 (0.59 – 1.05) | 0.108 |
| Allele model |  |  |  | 0.021* |  | 0.013* |
| T | 967 (57%) | 1027 (61%) | 1.00 |  | 1.00 |  |
| G | 725 (43%) | 655 (39%) | 0.85(0.74-0.98) |  | 0.83 (0.71 – 0.96) |  |
| Dominant model |  |  |  | 0.011* |  | 0.019* |
| T | 274 | 324 | 1.00 |  | 1.00 |  |
| GT+G | 573 | 523 | 0.77 (0.63-0.94) | 0.011* | 0.78 (0.63 - 0.96) | 0.019* |
| Recessive model |  |  |  | 0.334 |  | 0.906 |
| T+GT | 694 | 709 | 1.00 |  | 1.00 |  |
| G | 153 | 138 | 0.88 (0.69-1.14) | 0.334 | 0.98 (0.75 - 1.29) | 0.906 |
| **rs9925427A/G**  Genotypes |  |  |  | 0.902 |  | 0.826 |
| AA | 638 (77.2%) | 665 (79.1%) | 1.00 |  | 1.00 |  |
| GA | 208 (22.7%) | 192 (20.9%) | 0.95 (0.75-1.20) | 0.650 | 0.93 (0.73 – 1.18) | 0.537 |
| GG | 1 | 0 | 0.00 (0.00 - inf) | 0.969 | 0.00 (0.00-inf) | 0.969 |
| Allele model |  |  |  | 0.601 |  | 0.498 |
| A | 1459 (89%) | 1488 (89%) | 1.00 |  | 1.00 |  |
| G | 185 (11%) | 178 (11%) | 094 (0.76- 1.17) |  | 0.92 (0.73- 1.16) |  |
| Dominant model |  |  |  | 0.331 |  | 0.353 |
| A | 638 | 655 | 1.00 |  | 1.00 |  |
| GA+G | 209 | 192 | 0.89(0.72-1.12) | 0.331 | 0.90 (0.72 - 1.13) | 0.353 |
| Recessive model |  |  |  | 0.969 |  | 0.969 |
| A+GA | 846 | 847 | 1.00 |  | 1.00 |  |
| G | 1 | 0 | 0.00 (0.00-inf) | 0.969 | 0.00 (0.00-inf) | 0.969 |

^a^: adjust: gender、age、BMI、Hypertension、DM

*P<0.05
